# Derived karyotypes in two elephantfish genera (*Hyperopisus* and *Pollimyrus*): lowest chromosome number in the family Mormyridae (Osteoglossiformes)

**DOI:** 10.3897/compcytogen.v15.i4.67681

**Published:** 2021-10-08

**Authors:** Sergey Simanovsky, Dmitry Medvedev, Fekadu Tefera, Alexander Golubtsov

**Affiliations:** 1 Severtsov Institute of Ecology and Evolution, Russian Academy of Sciences, 33 Leninskij prosp., Moscow, 119071 Russia Severtsov Institute of Ecology and Evolution, Russian Academy of Sciences Moscow Russia; 2 National Fishery and Aquatic Life Research Center, Ethiopian Institute of Agricultural Research, Sebeta, P.O. Box 64, Ethiopia Ethiopian Institute of Agricultural Research Sebeta Ethiopia

**Keywords:** Africa, chromosomes, karyotype evolution, chromosome fusions, *Hyperopisus*, *Pollimyrus*

## Abstract

The African weakly electric elephantfish family Mormyridae comprises 22 genera and almost 230 species. Up-to-date cytogenetic information was available for 17 species representing 14 genera. Here we report chromosome number and morphology in *Hyperopisusbebe* (Lacepède, 1803) and *Pollimyrusisidori* (Valenciennes, 1847) collected from the White Nile system in southwestern Ethiopia. Both taxa displayed the diploid chromosome number 2n = 40, but they differed in fundamental numbers: FN = 66 in *H.bebe* and FN = 72 in *P.isidori*; previously the same diploid chromosome number 2n = 40 was reported in an undescribed species of *Pollimyrus* Taverne, 1971 (FN = 42) from the same region. Our results demonstrate that not only pericentric inversions, but fusions also played a substantial role in the evolution of the mormyrid karyotype structure. If the hypothesis that the karyotype structure with 2n = 50–52 and prevalence of the uni-armed chromosomes close to the ancestral condition for the family Mormyridae is correct, the most derived karyotype structures are found in the *Mormyrus* Linnaeus, 1758 species with 2n = 50 and the highest number of bi-armed elements in their compliments compared to all other mormyrids and in *Pollimyrusisidori* with the highest number of bi-armed elements among the mormyrids with 2n = 40.

## Introduction

The African weakly electric elephantfishes comprise the family Mormyridae including 22 genera and almost 230 species (Eschmeyer et al. 2021; Froese and Pauly 2021). To date, the representatives of 14 mormyrid genera have been studied cytogenetically (Uyeno 1973; Krysanov and Golubtsov 2014; Ozouf-Costaz et al. 2015; Canitz et al. 2016; Simanovsky et al. 2020, 2021). The diploid chromosome numbers in most elephantfishes vary between 48 and 52 with the mode 50 (Simanovsky et al. 2020). While a single studied species of the genus *Pollimyrus* Taverne, 1971 exhibited 2n = 40 (Krysanov and Golubtsov 2014).

A problem of the ancestral karyotype for the family Mormyridae was discussed by Canitz et al. (2016) and Simanovsky et al. (2020). In the first study, the most likely ancestral chromosome number for the family was identified as n = 24 or n = 25. In the latter study, three most parsimonious scenarios of the early karyotype evolution within the family were considered and the karyotype structure with 2n = 50–52 and prevalence of the uni-armed elements was suggested for a hypothetical ancestor. This suggestion was based on the following points. First, the family Mormyridae belongs to one of the most primitive groups of teleostean fishes, the cohort Osteoglossomorpha (Nelson et al. 2016), while the recent genomic data give evidence for the ancestral Euteleostomi karyotype of 50 chromosomes with domination by acrocentric elements (Nakatani et al. 2007; Sacerdot et al. 2018; de Oliveira et al. 2019). Second, for the family Notopteridae, the osteoglossomorph group closely related to mormyrids (Lavoué and Sullivan 2004, Nelson et al. 2016), the ancestral karyotype structure with 2n = 50 composed exclusively of uni-armed elements was suggested (Barby at al. 2018). Third, the karyotype structure with 2n = 50–52 and prevalence of the uni-armed elements is rather infrequent among mormyrids but appears in the genera displaying primitive morphology (mainly, dentition and electrocyte structure) and mainly basal phylogenetic positions (Taverne 1972; Alves-Gomes and Hopkins 1997; Sullivan et al. 2000).

Indeed, such karyotype structure is found in the two genera (*Petrocephalus* Marcusen, 1854 and *Mormyrops* Müller, 1843) appearing among the basal groups in molecular phylogenies of the family Mormyridae (Alves-Gomes and Hopkins 1997; Sullivan et al. 2000; Lavoué et al. 2003). The third basal genus (*Myomyrus* Boulenger, 1898) is not yet studied cytogenetically, while one more group with the seemingly primitive karyotype – *Stomatorhinuswalkeri* (Günther, 1867) (2n = 50, FN = 52) – does not display a basal position in the phylogenetic trees but its stemming is varying and poorly supported (Lavoué et al. 2003; Sullivan et al. 2016; Levin and Golubtsov 2018).

The karyotype structure with chromosome number unusually low for mormyrids was reported by Krysanov and Golubtsov (2014) for a representative of the genus *Pollimyrus*. This genus is among the most species-rich of mormyrid genera, and includes 19 species widely distributed throughout sub-Saharian Africa (Eschmeyer et al. 2021; Froese and Pauly 2021). Variation of the karyotype structure among the different *Pollimyrus* species has not been studied. The genus *Hyperopisus* Gill, 1862 not yet studied cytogenetically includes the only species *H.bebe* distributed in the Sahelo-Sudanese river basins (Eschmeyer et al. 2021; Froese and Pauly 2021). Both *Pollimyrus* and *Hyperopisus* never appeared among basal groups in the mormyrid molecular based phylogenies (Alves-Gomes and Hopkins 1997; Sullivan et al. 2000; Lavoué et al. 2003). Moreover, both genera exhibit some apparently derived morphological features related to the peculiarities of electrogeneration in *Pollimyrus* (Sullivan et al. 2000) and molluscivory in *Hyperopisus* (Taverne 1972; Bailye 1994).

In the present study, we address the uniqueness of the low chromosome numbers in mormyrids; *H.bebe* and the second species of the genus *Pollimyrus* were cytogenetically analyzed (for chromosome number and morphology). Based on the obtained and previous results, the two types of karyotype structure most derived from a hypothetical ancestral condition within the family Mormyridae were defined.

## Material and methods

Fishes were collected in Ethiopia within the framework of the Joint Ethiopian-Russian Biological Expedition (**JERBE**) with permission from the National Fishery and Aquatic Life Research Center under the Ethiopian Institute of Agricultural Research and the Ethiopian Ministry of Innovation and Technology. The experiments were carried out in accordance with the rules of the Severtsov Institute of Ecology and Evolution, Russian Academy of Sciences.

Three individuals (two females and a male) of each of the two species – *Hyperopisusbebe* (Lacepède, 1803) (standard length, SL 131–356 mm) and *Pollimyrusisidori* (Valenciennes, 1847) (SL 54–60 mm) – were karyotyped; total numbers of complete metaphase plates studied for each species were 30 and 33, respectively. Fish were sampled in the Gambela Peoples’ Region, a regional state in western Ethiopia at two sites in November of 2017: *P.isidori* from the Baro River downstream of the City of Itang (8°10'47"N, 34°15'2"E) and *H.bebe* from the Alvero River downstream of the Abobo Dam (7°52'23"N, 34°29'48"E). Both rivers belong to the Sobat River drainage discharging into the White Nile in South Sudan. Fish were caught with gill (*H.bebe*) and cast (*P.isidori*) nets, delivered in 80-l plastic containers into the field laboratory, where they were kept in permamently aerated water for several hours before treatment.

Before preparation fish were treated intraperitoneally with 0.1% colchicine for 3–4 hours. Then fish were euthanized with an overdose of tricaine methanesulfonate (MS-222), identified based on morphological key characters, measured to an accuracy of 1 mm, dissected for gonad examination and tissue sampling, and preserved in 10% formaldehyde. Vouchers are deposited at the Severtsov Institute of Ecology and Evolution (Moscow) under provisional labels of JERBE.

Chromosome preparations were obtained from anterior kidney according to Kligerman and Bloom (1977), procedures were described by Simanovsky and coauthors (2020, 2021). Giemsa-stained chromosome spreads were analysed under an “Axioplan 2 Imaging” microscope (Carl Zeiss, Germany) equipped with a “CV-M4+CL” camera (JAI, Japan) and “Ikaros” software (MetaSystems, Germany). Karyotypes were established according to the centromere position following the nomenclature of Levan et al. (1964). Chromosomes were classified as metacentric (m), submetacentric (sm) and acrocentric (a), including subtelocentric and telocentric chromosomes, and grouped according to their morphology in order of decreasing size. To determine the fundamental number (FN), metacentrics and submetacentrics were considered bi-armed and acrocentrics as uni-armed.

## Results and discussion

**Figure 1.**
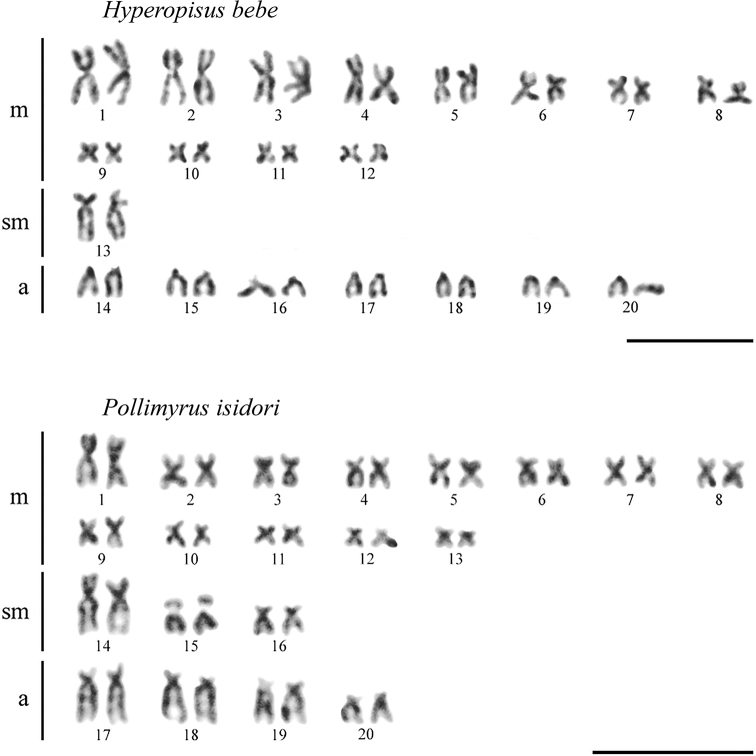
Karyotypes of *Hyperopisusbebe* and *Pollimyrusisidori* after conventional Giemsa staining. Scale bars: 10 μm.

*Hyperopisusbebe* has karyotype with 2n = 40 (Fig. 1) consisting of 24 metacentrics, 2 submetacentrics and 14 acrocentrics, the fundamental number FN = 66. *Pollimyrusisidori* has karyotype with 2n = 40 consisting of 26 metacentrics, 6 submetacentrics and 8 acrocentrics, FN = 72. In agreement with the lack of reports on sex chromosomes in other mormyrids, no distinguishable sex chromosomes were observed in complements of the two species.

For comparative purposes, all the currently available data on the karyotype structure in mormyrids are given in Table 1. Usage of the name Pollimyruspropenigricans (Boulenger, 1906) has been substantited by Krysanov and Golubtsov (2014). Division of the family Mormyridae into two subfamilies Petrocephalinae (including the single genus *Petrocephalus*) and Mormyrinae (including all other mormyrid genera), as well as usage of the names *Brienomyrusbrachyistius* (Gill, 1862), *Campylomormyrusrhynchophorus* (Boulenger, 1898) and *Paramormyrops* sp.7, have been discussed by Simanovsky et al. (2020). The karyotypes most similar to a hypothetical ancestral condition within the family based on arguments considered above are highlighted with bold in the Table 1.

**Table 1.** Cytogenetically studied elephantfishes of the family Mormyridae arranged in accordance with increasing (1) diploid chromosome number – 2n and (2) fundamental number – FN; karyotypic formulas most close to that in a hypothetic ancestor of the family are highlighted with bold.

Taxon	2n	Karyotypic formula	FN	Origin	References
**2n = 40**					
Pollimyruspropenigricans (Boulenger, 1906)	40	2m + 38a	42	White Nile and Omo-Turkana Basins, Ethiopia	Krysanov and Golubtsov 2014
*Hyperopisusbebe* (Lacepède, 1803)	40	24m + 2sm + 14a	66	White Nile Basin, Ethiopia	This study
*Pollimyrusisidori* (Valenciennes, 1847)	40	26m + 6sm + 8a	74	White Nile Basin, Ethiopia	This study
**2n = 48**					
*Brienomyrusbrachyistius* (Gill, 1862)	48	1m + 4sm + 2st + 41a	53	Unknown (fish store)	Uyeno 1973
*Brevimyrusniger* (Günther, 1866)	48	4m + 2sm + 42a	54	White Nile Basin, Ethiopia	Simanovsky et al. 2020
*Gnathonemuspetersii* (Günther, 1862)	48	10m + 6sm + 32a	64	Unknown (fish store)	Uyeno 1973
	48	18m + 2sm + 28a	68	Unknown (fish store)	Ozouf-Costaz et al. 2015
*Campylomormyrusrhynchophorus* (Boulenger, 1898)	48	26m + 4sm + 18a	78	Unknown (laboratory stock)	Canitz et al. 2016
**2n = 50**					
*Petrocephalusmicrophthalmus* Pellegrin, 1909	50	**2sm + 48a**	52	Ogooué Basin, Gabon	Ozouf-Costaz et al. 2015
*Stomatorhinuswalkeri* (Günther, 1867)	50	**2sm + 48a**	52	Ogooué Basin, Gabon	Ozouf-Costaz et al. 2015
*Marcuseniusmoorii* (Günther, 1867)	50	4sm + 46a	54	Ntem River, Gabon	Ozouf-Costaz et al. 2015
*Paramormyrops* sp.7	50	2m + 6sm + 42a	58	Woleu River, Gabon	Ozouf-Costaz et al. 2015
*Ivindomyrusopdenboschi* Taverne et Géry, 1975	50	10m + 2sm + 38a	62	Ntem River, Gabon	Ozouf-Costaz et al. 2015
*Cyphomyruspetherici* (Boulenger, 1898)	50	18m + 4sm + 28a	72	White Nile Basin, Ethiopia	Simanovsky et al. 2020
*Marcuseniuscyprinoides* (Linnaeus, 1758)	50	22m + 4sm + 24a	76	White Nile Basin, Ethiopia	Simanovsky et al. 2020
*Hippopotamyruspictus* (Marcusen, 1864)	50	24m + 4sm + 22a	78	White Nile Basin, Ethiopia	Simanovsky et al. 2020
*Mormyruscaschive* Linnaeus, 1758	50	20m + 14sm + 16a	84	White Nile Basin, Ethiopia	Simanovsky et al. 2021
*Mormyrushasselquistii* Valenciennes, 1847	50	20m + 14sm + 16a	84	White Nile Basin, Ethiopia	Simanovsky et al. 2021
*Mormyruskannume* Fabricius, 1775	50	20m + 14sm + 16a	84	Omo-Turkana Basin, Ethiopia	Simanovsky et al. 2021
**2n = 52**					
*Mormyropsanguilloides* (Linnaeus, 1758)	52	**52a**	52	White Nile Basin, Ethiopia	Simanovsky et al. 2020

The chromosome set of the undescribed species reported by Krysanov and Golubtsov (2014) as Pollimyruspropenigricans possessing 2n = 40 includes 2 small metacentric and 38 acrocentric chromosomes (FN = 42). Thus, despite the same diploid number of chromosomes (2n = 40), three taxa – *H.bebe* and two *Pollimyrus* species studied – display the substantially diverged structure of their karyotypes. Interestingly, two *Pollimyrus* species differ from each other in karyotype structure – mostly in the number of uni-armed elements – more than both from *H.bebe*. Judging from the molecular phylogenies (Lavoué et al. 2003; Sullivan et al. 2016; Levin and Golubtsov 2018), there is a possibility of independent reduction of the chromosome numbers in *Hyperopisus* and *Pollimyrus*. Eight studied species of the latter genus form a well supported monophyletic clade within the mormyrid tree, while the two *Pollimyrus* species analyzed cytogenetically are closely related (Levin and Golubtsov 2018). *Stomatorhinus* in some analyses appears as a sister group to the *Pollimyrus* clade, but the clade *Pollimyrus* + *Stomatorhinus* is poorly supported (Lavoué et al. 2003; Sullivan et al. 2016; Levin and Golubtsov 2018). The phylogenetic position of *Hyperopisus* is not resolved in any molecular phylogenetic studies. The unusually low number of chromosomes for mormyrids in this genus makes the question of its phylogenetic position even more intriguing.

*Pollimyrus* appears the third mormyrid genus for which the data on intrageneric variation of the karyotype structure are available (Table 1). In this genus the pronounced divergence between species is similar to the situation in *Marcusenius* Gill, 1862, where two species studied have the same diploid chromosome number, but different karyotypic formula – *M.moorii* (Günther, 1867) has 4sm + 46a, *M.cyprinoides* (Linnaeus, 1758) has 22m + 4sm + 24a (2n = 50 for both) (Ozouf-Costaz et al. 2015; Simanovsky et al. 2020). On the contrary, among three species of the genus *Mormyrus* Linnaeus, 1758 no difference in their karyotype structure was found (Simanovsky et al. 2021). Thus, a search for interspecific differences in the non-monotypic mormyrid genera looks quite informative.

Pericentric inversions are considered as the main type of chromosomal rearrangements in mormyrid karyotype evolution by Ozouf-Costaz et al. (2015). Finding of the three species with substantially reduced chromosome numbers (Table 1) indicates that fusions also played a substantial role in the evolution of the mormyrid karyotype structure. Along with the family Mormyridae, a substantial reduction of chromosome numbers seems to occur in the related lineages of the cohort Osteoglossomorpha. Very interesting data on *Gymnarchusniloticus* Cuvier, 1829, the only representative of the family Gymnarchidae and a sister group of Mormyridae, reveal unexpectedly different karyotype structures – 2n = 34 (26m + 8sm) and 2n = 54 (26m + 14sm + 14sta) – in the two Nigerian populations separated by a distance of less than 200 km (Hatanaka et al. 2018; Jegede et al. 2018). Notopteridae is a sister group of Mormyridae + Gymnarchidae (Lavoué and Sullivan 2004; Nelson et al. 2016). Concerning the only notopterid *Papyrocranusafer* (Günther, 1868) exhibiting karyotype with 2n = 50 (2m + 2sm + 46a), it was suggested that its diploid number remains unchanged compared to a hypothetical common ancestor of notopterids but the karyotype structure in *P.afer* is formed by intrachromosomal rearrangement of two chromosome pairs, resulting in bi-armed elements (Barby at al. 2018). The other notopterids possess exclusively uni-armed elements in their karyotype with 2n ranging from 38 to 46. For this group of taxa Barby at al. (2018) suggest the reduction of 2n via tandem fusions.

One may suggest that just tandem fusions played an important role in reduction of chromosome number to 2n = 40 at least in Pollimyruspropenigricans with FN = 42 (Table 1). Based on hypotheses about the dominating role of pericentric inversions in karyotype evolution in most other mormyrids (Ozouf-Costaz et al. 2015) and the ancestral karyotype structure with 2n = 50–52 and prevalence of the uni-armed chromosomes (Simanovsky et al. 2020), it is possible to consider the most parsimonious scenarios of an emergence of the karyotype diversity in the family. It is noteworthy that the karyotypes of all species with 2n = 50 could evolve from the ancestral karyotype with 2n = 50 and FN = 50 via pericentric inversions exclusively: from rearrangement of a single chromosome pair in *Petrocephalus* and *Stomatorhinus* to rearrangements of 17 chromosome pairs in *Mormyrus* Linnaeus, 1758 (Table 1). In our view, the karyotypes characterized by the lowest numbers of uni-armed elements may be considered as the most derived condition of the karyotype structure within the family. Particularly, based on the most parsimonious scenarios, the *Mormyrus* karyotype may be recognized as most derived among the mormyrids with 2n = 48–52, while the karyotype of *Pollimyrusisidori* seems to be most derived among the mormyrids with 2n = 40. Further studies with the use of more advanced cytogenetic techniques could verify the presented suggestions on the karyotype evolution within the family Mormyridae.
